# The Effects of Immediate Dentin Sealing on the Enzymatic Activity and Bond Strength of Hybrid Ceramic CAD/CAM Onlays: A Comparative Study of Two Universal Adhesives

**DOI:** 10.3390/dj14050281

**Published:** 2026-05-08

**Authors:** Uros Josic, Tatjana Maravic, Carlo D’Alessandro, Annamaria Forte, Diego D’Urso, Sofia Avnet, Edoardo Mancuso, Annalisa Mazzoni, Claudia Mazzitelli, Lorenzo Breschi

**Affiliations:** Department of Biomedical and Neuromotor Sciences, University of Bologna, 40125 Bologna, Italy; uros.josic2@unibo.it (U.J.); tatjana.maravic@unibo.it (T.M.); carlo.dalessandro5@unibo.it (C.D.); annamaria.forte2@unibo.it (A.F.); diego.durso2@unibo.it (D.D.); edoardo.mancuso@unibo.it (E.M.); annalisa.mazzoni@unibo.it (A.M.); claudia.mazzitelli@unibo.it (C.M.)

**Keywords:** immediate dentin sealing, luting, universal adhesive, universal resin composite cement

## Abstract

**Background/Objectives**: To elucidate the effects of immediate dentin sealing (IDS) with two universal adhesive systems on the microtensile bond strength (µTBS) and enzymatic activity (MMPs) of CAD/CAM hybrid ceramic onlays. **Methods**: Twenty-four human molars were assigned to one of the following groups (*n* = 8) according to IDS and the adhesives used: (1) Clearfil Universal Bond Quick (IDS QB; Kuraray); (2) Scotchbond Universal Plus (IDS SB; 3M); (3) no IDS (CTR). CAD/CAM onlays (Katana Avencia Block, Kuraray) were luted using a silane-containing universal composite cement (Panavia SA Cement Universal, Kuraray). µTBS tests and scanning electron microscope (SEM) analysis were performed after 24 h (T_0_) or 10,000 thermocycles (T_t_). Three additional molars per group were processed to evaluate the effects of IDS on MMPs using in situ zymography at T_0_ and T_t_. Data were statistically analyzed (α = 0.05). **Results**: At T_0_, IDS QB showed a significantly higher µTBS than IDS SB and CTR (*p* < 0.05). Artificial aging led to significant reductions in µTBS in IDS QB and CTR (*p* < 0.05), while µTBS remained stable in IDS SB. Both IDS groups demonstrated higher µTBS compared with CTR after aging (*p* < 0.05). At T_0_, the application of a universal adhesive system significantly increased the levels of MMPs (IDS QB > IDS SB > CTR; *p* < 0.05). At T_t_, IDS QB exhibited higher MMP activity compared with CTR (*p* < 0.05). **Conclusions**: IDS had a positive effect on immediate and aged µTBS, while the level of MMP activity was found to be material-dependent.

## 1. Introduction

Immediate dentin sealing (IDS), whose origins date back more than three decades [1], is a technique that has been increasingly adopted among dentists to improve the clinical performance of adhesively cemented indirect restorations and eliminate the initial postoperative sensitivity (POS) [2]. Although a chronological overview of the literature may reveal the evolution and different variants of the IDS technique, the concept of the originally proposed technique, referred to as the resin coating technique, first described in Japan [3], remains the same: immediately after a tooth has been prepared for an indirect restoration (partial or full-crown), a layer of an adhesive system is applied to the freshly cut and exposed dentin surface and then polymerized [4]. Unlike conventional delayed dentin sealing (DDS), where the adhesive is applied during the cementation procedure, the IDS technique protects the dentin from contamination with various provisional materials, bacteria and saliva during the provisional phase [1]. Additionally, applying and polymerizing the adhesive on freshly cut dentin promotes optimal hybridization and prevents collagen fibril collapse [5,6], leading to the formation of more “robust” hybrid layers and improved bond strength compared to the DDS technique [7]. Beyond reducing POS, IDS also contributes to better marginal adaptation of indirect restorations and ensures a more uniform adhesive interface between the dentin and restorative material [8,9].

The so-called “gold-standard” three-step etch-and-rinse (EAR) adhesive systems, such as Optibond FL (Kerr), were originally recommended for the IDS technique [4,10]. Two-step self-etch (SE) adhesives and, more recently, universal adhesive systems offer effective alternatives for dentin sealing, particularly for clinicians opting to avoid dentin etching [11,12]. The choice of adhesive system directly impacts the subsequent clinical procedures associated with the IDS technique. When using a three-step EAR adhesive system, applying a protective layer of flowable composite is not mandatory, as the IDS layer formed by this filled adhesive is resistant to air abrasion with aluminum oxide and to phosphoric acid etching during the cementation phase [12]. In contrast, when using unfilled or lightly filled adhesives, such as universal or certain SE systems, it is advisable to place a layer of flowable resin immediately after polymerizing the adhesive. This technique, known as “reinforced IDS” [13], provides protection against oxygen inhibition and helps to preserve the adhesive interface during temporary material removal, especially when air abrasion with aluminum oxide is performed [12]. However, some studies report that the layer of flowable composite has no influence on the bond strength when IDS is performed with simplified adhesive systems [14,15].

Recently, luting materials consisting of a resin composite cement and its dedicated adhesive system have been introduced to the dental market [16]. These cements are referred to as “universal” as they are indicated for the cementation of various materials and they can be coupled with their chemically compatible universal adhesive systems, which may improve bonding performance and, in some marketed cements, the degree of conversion in areas where light polymerization is difficult to achieve [17,18]. Recent literature reviews highlighted that universal resin composite cements show higher or similar bond strength regarding CAD/CAM or additively manufactured composite, ceramic, zirconia and metal substrates as compared to traditional primer-assisted/multistep and self-adhesive cements [19,20]. Interestingly, some of universal composite cements contain an incorporated silane coupling agent, which eliminates the need to apply a separate silane coupling agent without jeopardizing the bond strength [21].

The body of evidence supporting simplified luting procedures has increased; however, it remains unclear whether the bond strength of indirect restorations can be enhanced when IDS is performed using the universal adhesive recommended for a silane-containing universal resin composite cement. Furthermore, it is still unknown whether IDS performed with a non-dedicated universal adhesive (different from the manufacturer’s recommendations) can be safely combined with such cements for the luting of increasingly popular hybrid ceramic CAD/CAM restorations, which combine the positive characteristics of ceramics and resin-based polymer materials [22]. Endogenous (dentinal) enzymatic activity is crucial for understanding the degradation of the resin/dentin interface [23], as it can break down extracellular matrix components, such as dentin collagen [24]. Since the application of simplified universal adhesives may also activate matrix metalloproteinases (MMPs) [20], we aimed to investigate how two universal adhesives influence MMP activity when employed in the IDS technique—a topic that, to our knowledge, has not been previously investigated. Consequently, this laboratory study tested the following null hypotheses: (1) different universal adhesives used in the IDS technique do not improve the bonding performance of CAD/CAM hybrid ceramic onlays luted with a silane-containing universal cement immediately or after artificial aging; (2) IDS performed with different universal adhesives has no influence on MMP activity immediately or after artificial aging.

## 2. Materials and Methods

### 2.1. Specimen Preparation

The specimen preparation for bond strength testing was carried out by the same operator according to the guidelines of the Academy of Dental Materials (ADM) [25]. Freshly extracted intact human molars obtained from healthy adult donors after obtaining their informed consent and university ethical committee approval (protocol N°: 71/2019/OSS/AUSLBO) were used in the study. The middle–deep dentin was exposed by cutting the teeth perpendicularly to their long axis using a low-speed diamond saw (Microremet, Remet, Casalecchio di Reno, Italy) under water cooling. Subsequently, a standardized smear layer was created on the exposed dentin surface with #600-grit wet silicon carbide (SiC) paper [25]. The specimens were water-rinsed and gently dried with an oil-free air flow for 5 s, being careful not to desiccate the dentin surface. Specimens were then randomly assigned to the following groups (*n* = 8 per group, as recommended by the ADM guidelines [25]), according to the adhesive used for IDS:

Group IDS QB: Clearfil Universal Bond Quick (Kuraray, Noritake, Tokyo, Japan);

Group IDS SB: Scotchbond Universal Plus (3M, Neuss, Germany);

Group CTR: Control group (no IDS was performed).

In groups IDS QB and IDS SB, the adhesives were applied on the dentin surfaces in self-etch mode and polymerized with a light-emitting diode lamp (LED; 1470 mW/cm^2^, wavelength: 430–480 nm; Elipar Deep Cure, 3M, St. Paul, MN, USA), according to the manufacturers’ instructions. Next, the sealed dentin surface was covered with a glycerin gel and light-cured for an additional 10 s. The gel was then removed using a cotton pellet soaked with 70% ethyl alcohol [26]. A 3-mm-thick temporary restoration (Caviton, GC Corp., Tokyo, Japan) was then placed on the dentin surfaces in all groups, and the specimens were stored in artificial saliva at 37 °C for 1 week. The detailed compositions of the materials used, and their application modes, can be seen in Table 1.

Following the provisional phase, the material was mechanically removed using a dental explorer and the dentin surfaces were cleaned with a pumice–water slurry for 5 s at 500 rpm under 3.5× magnifying loupes. To simulate definitive cementation, 3-mm-thick CAD/CAM hybrid ceramic onlays (Katana™ AVENCIA™ Block, Kuraray, Tokyo, Japan) were air-abraded (0.2 MPa, 30 µm Al_2_O_3_) and cleaned in an ultrasonic bath (ethanol and water, 1:1 ratio) for 1 min. Next, the onlays were cemented using a silane-containing universal composite cement (PANAVIA™ SA Cement Universal, Kuraray, Tokyo, Japan) in self-adhesive mode under standardized seating pressure [27]. The cemented onlays were tack-cured for 2 s and the excess cement was removed using a dental explorer. After the final resin cement light-curing for an additional 40 s (Elipar Deep cure, 3M), the specimens were placed in artificial saliva and stored in an incubator at 37 °C for 24 h.

### 2.2. Microtensile Bond Strength (µTBS) Testing

After storage, the specimens were serially sectioned to obtain approximately 1-mm-thick sticks in accordance with the non-trimming technique of the microtensile bond strength (µTBS) test and stored in artificial saliva at 37 °C for 24 h. Afterwards, half of the specimens were subjected to µTBS testing after 24 h (T0), while the other half were subjected to artificial aging in a thermocycling machine (Tt: 10,000 cycles, 5–55 °C, dwell time 30 s; SD-Mechatronik, Westerham, Germany). The dimensions of each stick (ca. 0.9 mm × 0.9 mm × 8 mm) were measured using a digital caliper (±0.01 mm) and the bonded area was calculated for the subsequent conversion of µTBS values in Newtons (N) into units of stress (MPa). The sticks were stressed to failure using a testing machine (Bisco Inc., Schaumburg, IL, USA) at a crosshead speed of 1 mm/min. Failure mode analysis was performed using a stereomicroscope at 40× magnification (Stemi 2000-C; Carl Zeiss Jena GmbH, Jena, Germany). The failures were classified as follows: adhesive at the dentine/cement interface—AD; adhesive at the CAD/CAM ceramic restoration/cement interface—AC; cohesive—C (within dentin—CD, cement—CA or restoration—CC); and mixed—M (adhesive and cohesive failures occurred simultaneously).

### 2.3. Scanning Electron Microscope (SEM) Analysis of Fractured Interfaces

Representative fractured sticks from the μTBS test (*n* = 2 per group, with bond strength values close to the group mean) were used for the scanning electron microscope (SEM) analysis. Briefly, the sticks were fixed in a 2.5% glutaraldehyde in 0.1% cacodylate buffer solution and subsequently dehydrated in ascending ethanol concentrations (50%, 70%, 80%, 90%, 95%, 100%) and HMDS. The fractured sticks were then mounted on metal stubs and gold–palladium sputter-coated before evaluation at different magnifications (50×, 200×, 1000× at accelerating voltage of 15.00 kV).

### 2.4. In Situ Zymography Analysis

The in situ zymography analysis was performed in accordance with a previous study [28]. The middle–deep dentin of non-carious human molars (*n* = 3), based on previous studies [29,30], was exposed using a low-speed diamond saw under water cooling. Next, the cementation procedures were performed according to the previously defined groups, and the specimens were cut vertically into 1-mm-thick slabs to expose the resin/dentin interface or the hybrid layer. Half of the specimens were processed immediately for in situ zymography analysis, while the other half were subjected to thermocycling procedures as described above.

Each bonded slab with the exposed resin/dentin interface was glued to a microscope slide and ground down to obtain approximately 50-μm-thick specimens. In situ zymography was performed using quenched fluorescein-conjugated gelatin as the MMP substrate. The gelatin stock solution (diluted 1:8 with the dilution buffer and an anti-fading agent) was placed on top of each slab and covered with a coverslip. Slides were light-protected and incubated in a humidified chamber at 37 °C. Hydrolysis of the quenched fluorescein-conjugated gelatin substrate, indicative of endogenous gelatinolytic enzyme activity, was assessed by examination under a confocal microscope (Leica SP8, Leica Microsystems GmbH, Wetzlar, Germany; ex: 488 nm and em: lp530 nm). The hydrolysis of the quenched fluorescein-conjugated gelatin substrate, indicative of MMPs’ activity, was quantified as the integrated density of the fluorescence signals for each image using the ImageJ software (Version 2.9.0/1.53t, National Institutes of Health, Bethesda, MD, USA) by one operator, who was unaware of the group assignments.

### 2.5. Statistical Analysis

Since the data from the µTBS test and in situ zymography analyses were not normally and homogeneously distributed (failed Kolmogorov–Smirnov and modified Levene’s tests, respectively, *p* < 0.05; raw data available in Supplementary Material), the non-parametric Kruskal–Wallis and Dunn’s post hoc tests were run. A resin/dentin bonded stick was considered as a statistical unit, and a minimum of 15 sticks per tooth were obtained during µTBS testing. All analyses were performed by a statistician blinded to the groups using SigmaPlot 14.0 (Systat Software, Chicago, IL, USA). The significance level was set at α = 0.05.

## 3. Results

### 3.1. Microtensile Bond Strength (µTBS) Testing

The results from the µTBS test are presented in Table 2. The IDS QB group, which used a universal adhesive and resin cement from the same manufacturer, showed higher strength at T0 compared to the control and IDS SB groups (*p* < 0.001). No statistically significant difference (*p* = 0.138) was observed between the control group and the IDS SB group at T0. After thermocycling, the bond strength significantly decreased (*p* < 0.05) in the IDS QB and CTR groups. Both IDS groups demonstrated higher bond strength values compared to the CTR group (*p* < 0.05) after thermocycling.

### 3.2. Scanning Electron Microscope (SEM) Analysis of Fractured Interfaces

**Figure 1:**
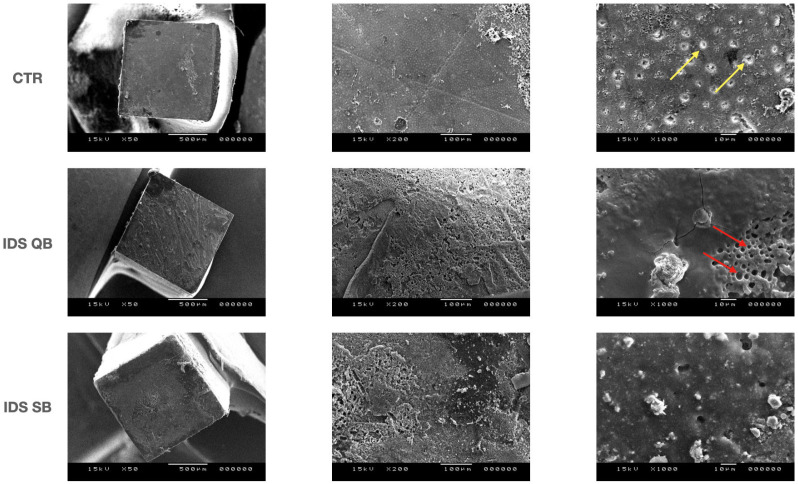
Scanning electron microscopy microphotographs (50×, 200× and 1000× magnifications) of the fractured specimens at baseline. Yellow arrows indicate dentinal tubules; red arrows indicate air bubbles.

**Figure 2:**
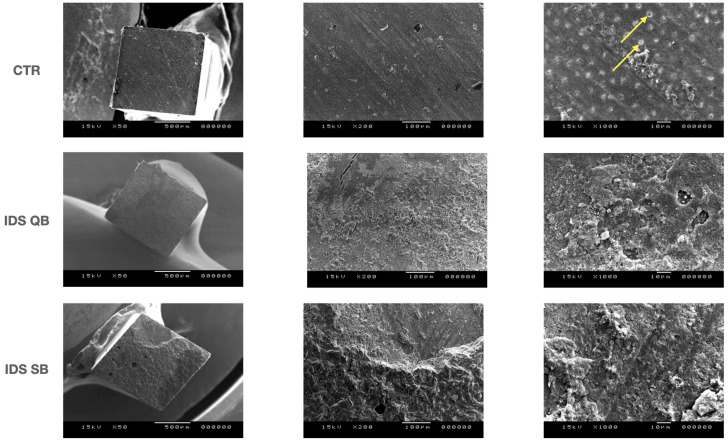
Scanning electron microscopy microphotographs (50×, 200× and 1000× magnifications) of the fractured specimens after artificial aging. Yellow arrows indicate dentinal tubules.

Representative SEM images (magnification 50×, 200× and 1000×, respectively) of the fractured specimens at T0 and Tt are shown in Figure 1 and Figure 2. In the CTR group, open dentinal tubules were clearly visible at T0, with the very limited presence of resin composite cement. On the other hand, the dentinal tubules were partially (QB) or mostly occluded (SB) in the IDS groups, with some air bubbles present in the IDS QB group. After thermocycling, the morphology of the fractured specimens in the CTR group remained largely unchanged. A rough surface with some microcracks was visible in the IDS QB group, while no exposed tubules could be seen in either of the IDS groups.

### 3.3. In Situ Zymography Analysis

**Figure 3:**
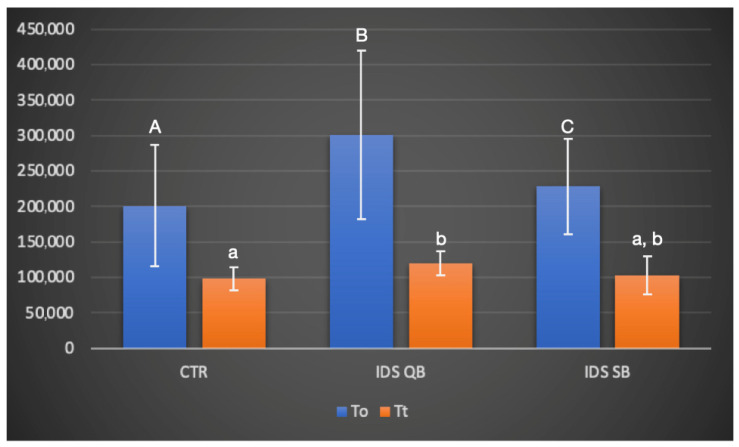
Gelatinolytic activity, expressed as a percentage of the green fluorescence within the resin/dentin interfaces created in the experimental groups, at baseline (T_0_) and after the thermocycling procedure (T_t_). Values are means and standard deviations. For a comparison of the factor “group”, columns that are labeled with different uppercase letters (T_0_) or lowercase letters (T_t_) are significantly different (*p* < 0.05).

**Figure 4:**
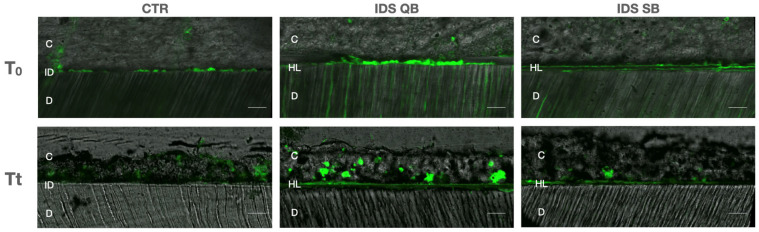
Confocal laser scanning microscopy images of resin/dentin interfaces that were incubated with quenched fluorescein-labeled gelatin in experimental groups at baseline (T_0_) and after the thermocycling procedure (T_t_). Abbreviations: C—composite (resin) cement; D—dentin; HL—hybrid layer; ID—interdiffusion zone. Scale bar: 100 micrometers.

Regarding the data retrieved from the in situ zymography analysis, at T0, significant differences in the levels of enzymatic dentinal activity between the groups (*p* < 0.05) were observed in the following order: IDS QB > IDS SB > CTR. After thermocycling, IDS QB showed a higher level of enzymatic activity compared to CTR (*p* = 0.022), while there were no other differences between the groups (Figure 3). Representative images of confocal laser scanning microscopy are presented in Figure 4. More intense fluorescence, alongside the hybrid layer and spreading towards dentinal tubules, was observed in the IDS QB group, while a low signal was observed in the CTR group. In general, there was a tendency toward a reduction in the fluorescence signal in all groups after thermocycling.

## 4. Discussion

**Figure 5:**
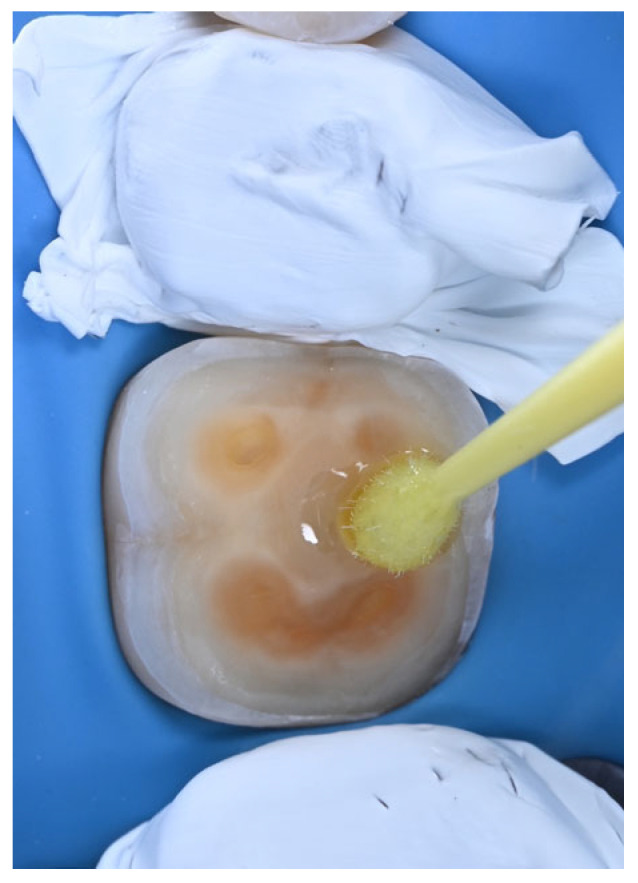
Application of a universal adhesive to freshly exposed dentin.

According to a recent literature review, IDS is an “elective clinical step that should be considered in patients and preparations with a higher risk of sensitivity between appointments” [31]. Due to their reduced technique sensitivity [24], universal adhesive systems used in self-etch mode may appear to be an attractive option for performing IDS (Figure 5). Given the wide variety of universal adhesives available on the dental market, the primary aim of this in vitro study was to investigate whether IDS performed with a universal adhesive system—other than the one specifically recommended by the cement manufacturer—could improve the bond strength of CAD/CAM hybrid ceramic indirect restorations luted with a silane-containing universal resin composite cement. The first null hypothesis was rejected, as the IDS QB group exhibited the highest baseline µTBS. After artificial aging, both IDS groups showed significantly higher µTBS than the CTR group, with all groups predominantly displaying mixed failure modes.

Both QB and SB are one-bottle universal adhesive systems that contain 10-methacryloyloxydecyl dihydrogen phosphate (10-MDP) as a functional monomer, with water and ethanol as solvents (data retrieved from patent literature) [20]. According to the revised adhesion–decalcification concept [32], the phosphoric acid ester functional group of 10-MDP chemically bonds to Ca of hydroxyapatite (HAP) within the non-demineralized dentin. Simultaneously, it also etches the HAP-based substrate, causing Ca release. Such Ca release causes the self-assembly of 10-MDP molecules into approximately 4 nm nanolayers, a process driven by the formation of stable 10-MDP-Ca salts, known as nanolayering [33]. Hybrid layers formed by the application of a 10-MDP-containing adhesive to unetched dentin benefit from both micromechanical interlocking and ionic bonding [34], resulting in favorable bond strength for resin-based restorations. The formation of hybrid layers following the application of QB and SB in the respective IDS groups is likely the mechanism responsible for the improved bond strength values after artificial aging observed in our study. Unlike in the IDS groups, the integrity of the CAD/CAM hybrid ceramic onlays in the CTR group relied solely on the capacity of the resin cement to etch and infiltrate the dentinal substrate [18]. A previous in vitro study reported that the application of a silane-containing universal composite cement to dentin in self-adhesive mode did not lead to the formation of “resin-like tag” structures visible on 1000× SEM micrographs [35], which explains the low luting potential of this material, as confirmed in our study.

Although both IDS groups demonstrated higher µTBS values compared to CTR after artificial aging, it is interesting to note that there were no baseline differences between the CTR and IDS SB groups. This suggests that the immediate beneficial effect of IDS in terms of bonding strength can be seen only when the manufacturer’s instructions are strictly followed, i.e., when a dedicated universal adhesive is coupled with its silane-containing resin cement. Nevertheless, the immediate µTBS values were not negatively influenced when a silane-containing cement was used in combination with a non-dedicated primer, a problem frequently reported with previous generations of composite cements and adhesive systems [20]. Indeed, although initially not different from the CTR group, the IDS SB group was the only one to maintain unchanged µTBS values after thermocycling, possibly due to the incorporation of the Vitrebond Copolymer, which further promotes ionic bonding during nanolayering [36]. Moreover, the lower level of MMP activity that was observed in the IDS SB group can explain the stable bond strength values.

The application of adhesive systems on dentin causes the activation of endogenous MMPs, leading to the degradation of hybrid layers and gradual loss of bond strength [37,38]. Higher enzymatic activity was observed in the IDS QB group at baseline, and this trend could be observed even after thermocycling, which led to the rejection of the second null hypothesis.

The greater activation of MMPs in the IDS QB group may possibly be attributed to compositional differences in the two adhesives, which are not always fully disclosed by the manufacturer. Even though the QB adhesive contains a newly developed amide monomer that should allow the rapid and enhanced penetration of the adhesive into dentin, the application of this adhesive according to the “no-wait” technique as advised by the manufacturer may have led to the creation of a suboptimal hybrid layer. In this case, the exposed collagen fibers may not have been entirely impregnated by the adhesive resin. Indeed, a previous laboratory study reported that, compared to SB, hybrid layers formed with QB were more susceptible to dye uptake within the adhesive interdiffusion zone and showed a significant reduction in bond strength after 10 months of aging in artificial saliva [38]. Subsequently, we cautiously hypothesize that the hybrid layer formed in the IDS QB group, with insufficiently impregnated collagen fibers, may have been more vulnerable to the activity of the MMPs over time. The higher initial enzymatic activity observed in the IDS QB group could be one of the mechanisms that was responsible for the reduction in the bond strength values after artificial aging [20]. Additionally, the “no-wait” application of adhesive, compared to the conventional “active scrubbing”, can lead to the formation of air bubbles, as shown in Figure 1, making the resin/dentin interfaces less stable after artificial aging. Interestingly, the attenuation of the fluorescence signal after thermocycling, corresponding to reduced MMP activity, has been reported previously [39,40], probably due to the fact that the distilled water used during thermocycling is free of the ions necessary for MMP activity [37]. However, this effect was generalized across all investigated groups, and therefore the comparisons between the groups are valid also at T_t_. Regardless of the reduction in the fluorescence signal after aging, it is evident that the initially elevated endogenous enzymatic activity, combined with temperature stresses (which may have led to substrate depletion and enzyme denaturation) and water uptake at the resin/dentin interface, followed by resin hydrolysis over 10,000 thermocycles, synergistically reduced the bond strength values in this study.

Although higher than the control group (no IDS), a previous study reported no statistically significant differences in µTBS values when Scotchbond Universal and Clearfil Universal Bond Quick were employed during IDS procedures [41]. Similarly, another study [42] showed that IDS achieved with the Scotchbond Universal adhesive without a protective layer composed of a flowable composite had no beneficial effect on the three-month µTBS values. These findings differ from ours probably due to the adhesive investigated in the previous studies (Scotchbond Universal), which is a predecessor of the adhesive used in this study (Scotchbond Universal Plus), when performing the IDS procedure. According to the manufacturer’s brochure, the latest version of the SB adhesive, the one used in the present study, contains an optimized mixture of silanes, is BPA derivate-free and contains a dual-cure accelerator (Vitrebond Copolymer) for improved bonding. Interestingly, a recent in vitro study that compared Scotchbond Universal to Scotchbond Universal Plus (SB) showed a narrower oxygen inhibition layer, with a higher hardness value, elastic modulus and degree of conversion in the latter material [43]. Furthermore, although not statistically different, when used in self-etch mode, SB did have higher µTBS compared to its predecessor—all of which could explain why the beneficial effect of IDS SB was observed in our study.

Methodological aspects that could influence the interpretation of our results should be addressed. The high standard deviations observed for the baseline μTBS values in the CTR and IDS QB groups are likely attributable to the heterogeneity of the dentin substrate; therefore, increasing the sample size in future studies may help to reduce this variability. Although this study demonstrated that IDS improved μTBS under standardized in vitro conditions, other factors may also influence bonding performance in clinical settings. These include the dentin substrate characteristics (e.g., presence of sclerotic dentin and tubule density), operator technique and the composition and application of the adhesive and resin cement, which may affect the relative contribution of IDS. Thermocycling, as a means of artificial aging, does not simulate pH fluctuations and mechanical fatigue, which should be introduced in future studies [44].

The use of temporary materials that are eugenol-free, such as the one used in this study, has been shown to have little or no effect on bond strength [8]. Although CAD/CAM hybrid ceramic onlays were used, the current study aimed to simulate a clinical scenario in which treatment could not be completed in a single visit. A similar, two-appointment approach was simulated in a recent study [45] that reported IDS as the preferred protocol when a tooth needs to be restored indirectly between the two visits. In general, it is considered that a one-week provisional phase has no negative effect on the bond strength values of indirect restorations when these specimens are stored in artificial saliva [10]. Additionally, the cleaning method seems to have a significant impact on the IDS layer. In this regard, less aggressive approaches, such as cleaning with pumice instead of air particle abrasion, have been reported as a suitable option for preserving the thin hybrid layers formed by universal adhesives [46]. This factor may explain why the IDS layer remained intact in our study. Lastly, to simulate a simplified clinical workflow, a silane-containing universal composite cement was used, simplifying the protocol by eliminating the need for additional pretreatment steps or adhesive re-application [47]. Future studies should investigate the impact of adhesive re-application during cementation, as this variable was not explored in the present study, and should also evaluate the effectiveness of the recently launched Clearfil Universal Bond Quick 2 material.

## 5. Conclusions

When luting CAD/CAM hybrid ceramic onlays with a silane-containing universal resin cement, immediate dentin sealing achieved with both tested universal adhesives had a positive impact on aged bond strength values. The combination of a silane-containing cement with a dedicated universal adhesive provided the highest baseline bond strength values. The activation of endogenous enzymatic activity was adhesive (material)-dependent and may have accounted for the reduction in bond strength after artificial aging.

## Figures and Tables

**Table 1:** Brands, manufacturers, chemical compositions, application protocols and batch numbers of the materials used in this study.

Material	Composition	Application Protocol	Batch Number
Clearfil Universal Bond Quick (Kuraray Noritake, Tokyo, Japan)	Bis-GMA ethanol HEMA 10-MDP Hydrophilic amide monomers Colloidal silica Silane coupling agent Sodium fluoride Camphorquinone Water	Apply in rubbing motion, no waiting, mildly air-dry (≥5 s) until the adhesive no longer moves. Light-cure for 10 s.	LOT220289
Scotchbond Universal Plus Adhesive (3M, Neuss, Germany)	Brominated dimethacrylate HEMA 10-MDP 2-Propenoic acid, 2-methyl-, 3-(triethoxysilyl) propylester, reaction products with silica and 3-(triethoxysilyl)-1-propanamine Ethanol Water Synthetic amorphous silica, fumed, crystalline-free γMPTES camphorquinone Copolymer of acrylic and itaconic acid N,n-dimethylbenzocaine APTES DEGDMA Acetic acid, copper(2+) salt, monohydrate	Apply a thin layer to the adhesive substrate with a micro-tip applicator and rub for 20 s. Air-blow gently for 5 s. Light-cure for 10 s.	LOT8101151
PANAVIA SA Cement Universal (Kuraray Noritake, Tokyo, Japan)	Paste A: 10-MDP, bis-GMA, TEG-DMA, hydrophobic aromatic dimethacrylate, HEMA, silanated barium glass filler, silanated colloidal silica, camphorquinone, peroxide, catalysts, pigments Paste B: hydrophobic aromatic dimethacrylate, silane coupling agent, silanated barium glass filler, aluminum oxide filler, surface-treated sodium fluoride, camphorquinone, accelerators, pigments	Before the first use, let a small amount bleed out and discard it. Attach a mixing tip, extrude a small amount, then apply the material on the surfaces to be luted. Light-cure for 2 to 5 s or let self-cure for 2 to 4 min, then remove the excess cement.	LOT830018

**Table 2:** Microtensile bond strength values (MPa) with means and standard deviations after 24 h of artificial saliva storage (T_0_) and after 10,000 thermocycles (T_t_). Different superscript lowercase letters indicate differences within the rows; different superscript uppercase letters indicate differences within the columns. The percentages of different failure modes are presented in parentheses under each respective bond strength value. AD—adhesive at the dentin interface; AC—adhesive at the CAD/CAM ceramic restoration interface; cohesive—C (within dentin—CD, cement—CA or restoration—CC); M—mixed failure.

Group	T0	Tt
IDS QB	12.7 ± 6.0 ^A,a^ (88M/8AD/4AC/0C)	8.0 ± 3.6 ^A,b^ (96M/4AD/0AC/0C)
IDS SB	5.7 ± 0.8 ^B,a^ (88M/3AD/9AC/0C)	6.6 ± 0.6 ^A,a^ (94M/6AD/0AC/0C)
CTR	8.3 ± 4.5 ^B,a^ (73M/19AD/8AC/0C)	3.4 ± 1.6 ^B,b^ (83M/17AD/0AC/0C)

## Data Availability

The original contributions presented in this study are included in the article/Supplementary Material. Further inquiries can be directed to the corresponding author.

## Supplementary Materials

The following supporting information can be downloaded at: https://www.mdpi.com/article/10.3390/dj14050281/s1, File S1: Raw data.

## References

[1] Pashley E.L., Comer R.W., Simpson M.D., Horner J.A., Pashley D.H., Caughman W.F. (1992). Dentin Permeability: Sealing the Dentin in Crown Preparations. Oper. Dent..

[2] Alghauli M.A., Alqutaibi A.Y., Borzangy S. (2025). Clinical Benefits of Immediate Dentin Sealing: A Systematic Review and Meta-Analysis. J. Prosthet. Dent..

[3] Nikaido T., Tagami J., Yatani H., Ohkubo C., Nihei T., Koizumi H., Maseki T., Nishiyama Y., Takigawa T., Tsubota Y. (2018). Concept and Clinical Application of the Resin-Coating Technique for Indirect Restorations. Dent. Mater. J..

[4] Magne P. (2005). Immediate Dentin Sealing: A Fundamental Procedure for Indirect Bonded Restorations. J. Esthet. Restor. Dent..

[5] Breschi L., Josic U., Maravic T., Mancuso E., Del Bianco F., Baldissara P., Mazzoni A., Mazzitelli C. (2023). Selective Adhesive Luting: A Novel Technique for Improving Adhesion Achieved by Universal Resin Cements. J. Esthet. Restor. Dent..

[6] Carvalho M.A., Lazari-Carvalho P.C., Maffra P.E.T., Izelli T.F., Gresnigt M., Estrela C., Magne P. (2025). Immediate Pre-Endodontic Dentin Sealing (IPDS) Improves Resin-Dentin Bond Strength. J. Esthet. Restor. Dent..

[7] Magne P., Kim T.H., Cascione D., Donovan T.E. (2005). Immediate Dentin Sealing Improves Bond Strength of Indirect Restorations. J. Prosthet. Dent..

[8] Elbishari H., Elsubeihi E.S., Alkhoujah T., Elsubeihi H.E. (2021). Substantial In-Vitro and Emerging Clinical Evidence Supporting Immediate Dentin Sealing. Jpn. Dent. Sci. Rev..

[9] Moghaddas M.J., Majidinia S., Shahri A., Shooshtari Z., Shirazi A.S., Borouziniat A., Azizi Z. (2026). The Effect of Immediate Dentin Sealing on the Marginal Adaptation and Microleakage of Indirect Restorations: A Systematic Review and Meta-Analysis. Int. Dent. J..

[10] Magne P., So W.-S., Cascione D. (2007). Immediate Dentin Sealing Supports Delayed Restoration Placement. J. Prosthet. Dent..

[11] Duarte S., de Freitas C.R.B., Saad J.R.C., Sadan A. (2009). The Effect of Immediate Dentin Sealing on the Marginal Adaptation and Bond Strengths of Total-Etch and Self-Etch Adhesives. J. Prosthet. Dent..

[12] De Carvalho M.A., Lazari-Carvalho P.C., Polonial I.F., de Souza J.B., Magne P. (2021). Significance of Immediate Dentin Sealing and Flowable Resin Coating Reinforcement for Unfilled/Lightly Filled Adhesive Systems. J. Esthet. Restor. Dent..

[13] Varadan P., Balaji L., Manaswini D.Y., Rajan R.M. (2022). Reinforced Immediate Dentin Sealing vs Conventional Immediate Dentin Sealing on Adhesive Behavior of Indirect Restorations: A Systematic Review. J. Contemp. Dent. Pract..

[14] Yılmaz Atalı P., Türkmen C., Günsel Kesimli E. (2021). Effect of Immediat Dentin Sealing on the Bonding State of Hybrid Ceramic CAD/CAM Restorative Material to Dentin. Eur. J. Res. Dent..

[15] Tahoun F.-A.M., Kehela H.A.-G., Nasr D.M. (2024). Influence of Different Immediate Dentin Sealing Strategies on Bond Strength of Indirect Resin Nanoceramic Restorations. Eur. J. Oral Sci..

[16] Lorey T., Woolford J., Blatz M.B., Lohbauer U., Belli R., Zorzin J.I. (2026). A Novel Method to Assess the Interfacial Fracture Toughness of Two Universal Resin Cements to Dentin. Dent. Mater..

[17] Tang C., Mercelis B., Ahmed M.H., Yoshihara K., Peumans M., Van Meerbeek B. (2023). Adhesive Performance Assessment of Universal Adhesives and Universal Adhesive/Composite Cement Combinations. J. Adhes. Dent..

[18] Maravić T., Mazzitelli C., Mancuso E., Del Bianco F., Josić U., Cadenaro M., Breschi L., Mazzoni A. (2023). Resin Composite Cements: Current Status and a Novel Classification Proposal. J. Esthet. Restor. Dent..

[19] de Carvalho S.B., Uehara L.M., Carvalho-Silva J.M., dos Reis A.C. (2026). Bonding Effectiveness of 10-MDP Containing Resin Composite Cements: A Systematic Review with Meta-Analysis. Int. J. Adhes. Adhes..

[20] Breschi L. (2025). Buonocore Memorial Lecture 2023: Changing Operative Mindsets with Universal Adhesives and Cements. Oper. Dent..

[21] Yoshihara K., Nagaoka N., Maruo Y., Nishigawa G., Yoshida Y., Van Meerbeek B. (2020). Silane-Coupling Effect of a Silane-Containing Self-Adhesive Composite Cement. Dent. Mater..

[22] Duarte S., Sartori N., Phark J.-H. (2016). Ceramic-Reinforced Polymers: CAD/CAM Hybrid Restorative Materials. Curr. Oral Health Rep..

[23] Anumula L., Ramesh S., Kolaparthi V.S.K. (2024). Matrix Metalloproteinases in Dentin: Assessing Their Presence, Activity, and Inhibitors—A Review of Current Trends. Dent. Mater..

[24] Vidal C.M.P., Carrilho M.R. (2023). Dentin Degradation: From Tissue Breakdown to Possibilities for Therapeutic Intervention. Curr. Oral Health Rep..

[25] Armstrong S., Breschi L., Özcan M., Pfefferkorn F., Ferrari M., Van Meerbeek B. (2017). Academy of Dental Materials Guidance on in Vitro Testing of Dental Composite Bonding Effectiveness to Dentin/Enamel Using Micro-Tensile Bond Strength (ΜTBS) Approach. Dent. Mater..

[26] Pheerarangsikul N., Wayakanon P., Wayakanon K. (2022). Effects of Various Functional Monomers on Adhesion Between Immediate Dentin Sealing and Resin Cement. Oper. Dent..

[27] Mancuso E., Gasperini T., Maravic T., Mazzitelli C., Josic U., Forte A., Pitta J., Mazzoni A., Fehmer V., Breschi L. (2024). The Influence of Finishing Line and Luting Material Selection on the Seating Accuracy of CAD/CAM Indirect Composite Restorations. J. Dent..

[28] Mazzoni A., Nascimento F.D., Carrilho M., Tersariol I., Papa V., Tjäderhane L., Di Lenarda R., Tay F.R., Pashley D.H., Breschi L. (2012). MMP Activity in the Hybrid Layer Detected with *in Situ* Zymography. J. Dent. Res..

[29] Maravic T., Breschi L., Paganelli F., Bonetti G., Martina S., Di Giorgio G., Bossù M., Polimeni A., Checchi V., Generali L. (2021). Endogenous Enzymatic Activity of Primary and Permanent Dentine. Materials.

[30] Er-Vin W., Marn Y.C., Shuai W.S., Singh J.K.A./P.K., Rehman K., Sidhu P., Thou L.C., Tang A.A., Ying N.H., Mahdi S.S. (2025). Synergistic Strategy of Riboflavin and Lipoids to Bioengineer Resin Dentin Hybrid Layer. Sci. Rep..

[31] Portella F.F., Müller R., Zimmer R., Reston E.G., Arossi G.A. (2024). Is Immediate Dentin Sealing a Mandatory or Optional Clinical Step for Indirect Restorations?. J. Esthet. Restor. Dent..

[32] Van Meerbeek B., Yoshihara K., Van Landuyt K., Yoshida Y., Peumans M. (2020). From Buonocore’s Pioneering Acid-Etch Technique to Self-Adhering Restoratives. A Status Perspective of Rapidly Advancing Dental Adhesive Technology. J. Adhes. Dent..

[33] Ahmed M.H., Esteban D.A., Attik N., Awad M.M. (2025). Nanomechanical Interlocking Mechanism of 10-MDP Nanolayering. Sci. Rep..

[34] Fehrenbach J., Isolan C.P., Münchow E.A. (2021). Is the Presence of 10-MDP Associated to Higher Bonding Performance for Self-Etching Adhesive Systems? A Meta-Analysis of in Vitro Studies. Dent. Mater..

[35] Abdel-Gawad S., Dursun E., Ceinos R., Le Goff S., Fasham T., Attal J.-P., Francois P. (2024). Touch-Cure Activation by Marketed Universal Resin Luting Cements of Their Associated Primer to Dentin. J. Oral Sci..

[36] Sezinando A., Serrano M.L., Pérez V.M., Muñoz R.A.G., Ceballos L., Perdigão J. (2016). Chemical Adhesion of Polyalkenoate-Based Adhesives to Hydroxyapatite. J. Adhes. Dent..

[37] Josic U., Mazzitelli C., Maravic T., Comba A., Cadenaro M., Radovic I., Sebold M., Turco G., Breschi L., Mazzoni A. (2023). The Effect of Carbodiimide on Push-out Bond Strength of Fiber Posts and Endogenous Enzymatic Activity. BMC Oral Health.

[38] Maciel Pires P., Dávila-Sánchez A., Faus-Matoses V., Nuñez Martí J.M., Lo Muzio L., Sauro S. (2022). Bonding Performance and Ultramorphology of the Resin-Dentine Interface of Contemporary Universal Adhesives. Clin. Oral Investig..

[39] Nisar S., Liu H., Hass V., Wang Y. (2023). Dual-Functional Etchants That Simultaneously Demineralize and Stabilize Dentin Render Collagen Resistant to Degradation for Resin Bonding. Dent. Mater..

[40] Chou Y.-F., Maciel Pires P., D’Urso D., Ozan G., Mazzitelli C., Maravic T., Sancaklı H.Ş., Breschi L., Sauro S. (2025). Effects of a Biomimetic Dual-Analogue Primer on the Bonding Performance of an Experimental Ion-Releasing Adhesive System—An in Vitro Study. J. Dent..

[41] Fazlioglu L., Oglakci Ozkoc B., Tagtekin D. (2025). The Effect of Immediate Dentin Sealing Using Different Universal Adhesives on the Bond Strength of Pretreated Monolithic Zirconia to Dentin and Microscopic Morphological Alterations. Microsc. Res. Tech..

[42] Batista J., Leite M.M., Sabag M.F., Lopes L.G., Torres É.M. (2022). Influence of the Flowable Resin Layer on Bond Strength Between Resin Cement and a Universal Adhesive Applied in the Immediate Dentin-Sealing Technique. Oper. Dent..

[43] Alam A., Yamauti M., Chowdhury A.F.M.A., Wang X., Álvarez-Lloret P., Zuñiga-Heredia E.-E., Cifuentes-Jiménez C., Dua R., Iijima M., Sano H. (2024). Evaluating the Advancements in a Recently Introduced Universal Adhesive Compared to Its Predecessor. J. Dent. Sci..

[44] De Munck J., Van Landuyt K., Peumans M., Poitevin A., Lambrechts P., Braem M., Van Meerbeek B. (2005). A Critical Review of the Durability of Adhesion to Tooth Tissue: Methods and Results. J. Dent. Res..

[45] Ramos R.Q., Mercelis B., Ahmed M.H., dos Santos B.C., Lopes G.C., Van Meerbeek B., Peumans M. (2025). Impact of Immediate versus Delayed Dentin Sealing on Adhesive Luting Effectiveness. Dent. Mater..

[46] Ozer F., Batu Eken Z., Hao J., Tuloglu N., Blatz M.B. (2024). Effect of Immediate Dentin Sealing on the Bonding Performance of Indirect Restorations: A Systematic Review. Biomimetics.

[47] Willers A.E., Gomes Araújo-Neto V., Bosso André C., Giannini M. (2024). Bond Durability of a Silane-Containing Self-Adhesive and Conventional Resin Cements to CAD/CAM Glass and Hybrid Ceramics. J. Adhes. Sci. Technol..

